# The WHO position on rabies immunization – 2018 updates

**DOI:** 10.1016/j.vaccine.2018.10.014

**Published:** 2019-10-03

**Authors:** Katherine L. O'Brien, Terry Nolan, on behalf of the SAGE WG on Rabies

**Affiliations:** a Johns Hopkins Bloomberg School of Public Health, USA; b Melbourne School of Population and Global Health, University of Melbourne, Australia

**Keywords:** Rabies, SAGE, Vaccine, WHO, Immunoglobulin

## 1 Rabies is fatal but preventable

Rabies is a neglected zoonotic disease responsible for an estimated 59,000 human deaths annually [1]. Rural populations in Africa and Asia are predominantly affected, and approximately 40% of cases occur in children under the age of 15 years. Transmitted through bites and scratches from infected animals, dogs are responsible for up to 99% of human rabies cases [2]. Although fatal once clinical signs appear, rabies is preventable through (i) mass dog vaccination to control disease at its source; (ii) awareness of rabies and the need to seek treatment if exposed; (iii) timely post-exposure prophylaxis (PEP) for people potentially exposed to rabies; and (iv) pre-exposure prophylaxis (PrEP) for those at high risk of rabies virus exposure.

PEP is administered after a potential exposure to rabies virus, and consists of rigorous wound washing, a series of rabies vaccination, and sometimes administration of rabies immunoglobulin (RIG) (Table 1). RIG can be derived from equine (eRIG), human (hRIG), or monoclonal antibody (mAb) sources [3]. PrEP consists of a series of rabies vaccination administered prior to a potential exposure. PrEP is recommended for individuals at high risk of rabies exposure, such as those at occupational risk, sub-populations in highly endemic settings who lack access to timely and adequate PEP, and travellers who may be at high risk of exposure [3]. Although PEP and PrEP can be administered intramuscularly (IM) or intradermally (ID), ID vaccination is both dose and cost sparing [4]. Modern purified cell-culture and embryonated egg-based rabies vaccines are highly immunogenic, effective, and safe to use in people of all ages [3].

**Table 1:** WHO categories of rabies exposure and indications for PEP [3].

WHO category of rabies exposure	PEP indications
Category I (i.e. no exposure): touching or feeding an animal, licks on intact skin	PEP not indicated
Category II (i.e. exposure): minor scratches or abrasions without bleeding	PEP indicated (wound washing and vaccine only) Treat as category III if exposure was to a bat
Category III (i.e. severe exposure): single or multiple transdermal bites or scratches, contamination of mucous membrane or broken skin with saliva from animal licks, exposures due to direct contact with bats	PEP indicated (wound washing, vaccine and RIG)

## 2 2018 update to WHO recommendations on rabies prophylaxis

The Strategic Advisory Group of Experts on Immunization (SAGE) established a Working Group (WG) on rabies vaccines and immunoglobulins in 2017 to undertake a comprehensive review of evidence and to propose revisions to SAGE on recommended rabies prophylaxis [5], [6]. This represents the first set of rabies immunization recommendations developed through the systematic working group process and replaces the 2010 recommendations. The 2018 update of the WHO position on rabies vaccines responds to the need for more programmatically feasible recommendations that aim to improve public health outcomes for rabies while maintaining the highest level of individual efficacy [5]. By addressing the discrepancy between previous WHO recommendations and current practices of PEP and PrEP usage in endemic areas, the update aims to consider the most recent evidence available to improve access to life-saving care for vulnerable populations. This includes a focus on improving delivery of rabies PEP to better meet the needs of underserved populations through shorter, less costly and more feasible PEP and PrEP protocols, and for prudent use of RIG, without compromising effectiveness. The revised WHO position complements other, parallel efforts to provide clear and practical guidance for rabies prevention, such as the 3rd WHO Expert Consultation on Rabies [2], the ongoing updates to the WHO diagnostic manual on Laboratory Techniques in Rabies, and the Global Strategic Plan to End Human Deaths from Dog-Mediated Rabies by 2030 [7].

## 3 Key changes to the WHO rabies immunization position

Key changes to the WHO rabies position are summarized below; the 2018 rabies position paper provides detailed descriptions of the literature and the recommendations [3].

### 3.1 Summary of updated recommendations for PEP and PrEP [3]

Evidence shows that modern rabies vaccines (>2.5 IU/IM dose), when administered ID for either PEP or PrEP, have immunogenicity and effectiveness equivalent to or higher than IM administration [8]. When administered via the ID route, 0.1 ml of CCEEV is used, and when administered IM a full vial is used (0.5 ml or 1 ml) for each dose. Rabies vaccines and RIG are considered safe to use in pregnant and lactating women, HIV-infected and other potentially immunocompromised individuals.

Vigorous wound washing with soap, detergent and copious amounts of water should be performed immediately, or as soon as possible, for all bites, scratches, and mucosal exposures. Previously WHO-recommended rabies vaccine schedules remain acceptable, however WHO now also recommends newer, shorter vaccine regimens that reduce costs, quantity of vaccine, and number of clinic visits required for both PEP and PrEP. These new PEP schedules for immunologically naïve individuals (all age groups) include (a) 2-site ID on days 0, 3 and 7 or (b) 1-site IM on days 0, 3, 7 and a final dose between days 14–28.

RIG is indicated in category III exposures for immunologically naïve individuals. Regardless of RIG availability, all category III exposed patients should receive rabies vaccines immediately. RIG should be administered only once, preferably at initiation of PEP and not more than 7 days following the first rabies vaccine dose. Skin testing prior to eRIG administration is unreliable and should not be performed. If available, rabies mAb products provide a potential alternative to RIG.

If a limited amount of RIG is available, its allocation should be prioritised for patients with high risk, category III exposures: multiple bites; those with deep wounds, or bites to highly innervated parts of the body, such as the head, neck and hands; patients with severe immunodeficiency; and cases where the biting animal is a confirmed or probable rabies case, or where bites, scratches or exposure of a mucous membrane were caused by a bat.

RIG should be infiltrated into and around the wound up to the maximum calculated volume^1^. If the wounds are large or multiple, the maximum calculated volume of RIG can be diluted with physiological buffered saline to allow sufficient volume for complete wound infiltration. By contrast, if the calculated RIG dose is more than can be fully injected in and around the wound itself, WHO no longer recommends injecting the remainder of the calculated RIG dose IM at a site distant from the wound. Therefore, if the calculated RIG dose^1^ is likely too great for local wound infiltration, it can be fractionated into smaller, individual syringes and the residual unused RIG can be used that same day for other patients, if stored and handled aseptically. Unused, fractionated RIG should be discarded at the end of the day.

PrEP should only be considered for persons at high risk of rabies exposure (see position paper) [3]. Individuals with documented evidence of previous PrEP, or at least 2 previous administrations of rabies vaccine as PEP, are considered previously immunized and benefit from an abridged PEP without RIG in case of exposure. The PrEP schedules that are now recommended for people in all age groups are (a) 2-site ID on days 0 and 7, or (b) 1-site IM on days 0 and 7.

### 3.2 Future rabies vaccine research needs

The rabies working group review process identified gaps in methodological guidelines to assess non-inferiority of new rabies PEP and PrEP regimens, factors determining clinical outcomes in immunocompromised individuals, and evidence-based best practice for repeat exposures. It highlighted the need for new vaccines with improved thermostability, prolonged shelf-life and reduced packaging volume; and novel tools to improve delivery at the community level; and vaccines with enhanced profiles that will be protective against lyssaviruses other than rabies virus. Further evidence is needed on the potential for even shorter PEP and PrEP regimens, such as one-visit PrEP in rabies endemic settings (including special populations).

## 4 Public health impact of updated rabies immunization recommendations

Overall, the revised recommendations will make rabies vaccine schedules more efficient by allowing for the use of shorter, cost and dose saving ID regimens, and reducing the need for RIG (Table 2) [9]. The challenge is now for countries and communities to implement these changes to improve access to life-saving PEP and reduce the burden of this fatal disease.

**Table 2:** Overview of key changes in the 2018 update of the WHO rabies vaccine position paper.

Topic	2010 position paper	2018 position paper
PEP regimen duration	3–4 weeks 4–5 visits	1–2 weeks 3–4 visits
PrEP regimen duration	3–4 weeks 3 visits	1 week 2 visits
Vaccine dose PEP	ID: 0.8 ml IM: 5 ml	ID: 0.6 ml IM: 4 ml
RIG infiltration mode	Full calculated volume in and around wound. Residual volume IM, at distant site.	In and around wound only, up to maximum calculated volume. No IM administration.
RIG allocation	All category III exposures	High risk category III exposures

Adopting the new regimens, including switching from IM to ID vaccination is key to increasing affordability, ease, and access to rabies PEP and PrEP. Vaccine manufacturers are strongly encouraged to submit a license variation application to national regulatory authorities for inclusion of ID administration and WHO recommended schedules as approved use on the label.

Further, the development, licensure, and use of mAb products as an alternative to eRIG or hRIG is encouraged, as mAbs can be produced with standardized quality in large quantities, do not use animals in the production process, and have higher effectiveness and reduced risk of adverse events. To date, a single mAb product has been licensed in 2017 [5]. The development of products containing two or more mAbs with non-overlapping epitopes would be useful. Additionally, WHO recommends that a registry be maintained to monitor the post-licensure effectiveness of mAb products.

## 5 Conclusion

The 2018 update of the WHO position on rabies immunization is an important step towards improving public health outcomes for rabies, increasing health equity and ultimately reaching the global goal of zero human rabies deaths by 2030, worldwide. Countries are now tasked with implementing these recommendations to save lives, and help advance broader efforts to eliminate deaths from this vaccine-preventable disease.

## Footnotes

1 The maximum dose for RIG is 40 IU/kg bodyweight for equine RIG and F(ab)’2 products, and 20 IU/kg bodyweight for human RIG.
